# Vascular endothelial growth factor regulates melanoma cell adhesion and growth in the bone marrow microenvironment via tumor cyclooxygenase-2

**DOI:** 10.1186/1479-5876-9-142

**Published:** 2011-08-25

**Authors:** María Valcárcel, Lorea Mendoza, José-Julio Hernández, Teresa Carrascal, Clarisa Salado, Olatz Crende, Fernando Vidal-Vanaclocha

**Affiliations:** 1 Innoprot SL, Bizkaia Technology Park, Derio, Bizkaia, Spain; 2 Basque Country University School of Medicine and Dentistry, Department of Cellular Biology and Histology, Leioa, Bizkaia, Spain; 3 CEU-San Pablo University School of Medicine and Hospital of Madrid Scientific Foundation, Institute of Applied Molecular Medicine (IMMA), Madrid, Spain

## Abstract

**Background:**

Human melanoma frequently colonizes bone marrow (BM) since its earliest stage of systemic dissemination, prior to clinical metastasis occurrence. However, how melanoma cell adhesion and proliferation mechanisms are regulated within bone marrow stromal cell (BMSC) microenvironment remain unclear. Consistent with the prometastatic role of inflammatory and angiogenic factors, several studies have reported elevated levels of cyclooxygenase-2 (COX-2) in melanoma although its pathogenic role in bone marrow melanoma metastasis is unknown.

**Methods:**

Herein we analyzed the effect of cyclooxygenase-2 (COX-2) inhibitor celecoxib in a model of generalized BM dissemination of left cardiac ventricle-injected B16 melanoma (B16M) cells into healthy and bacterial endotoxin lipopolysaccharide (LPS)-pretreated mice to induce inflammation. In addition, B16M and human A375 melanoma (A375M) cells were exposed to conditioned media from basal and LPS-treated primary cultured murine and human BMSCs, and the contribution of COX-2 to the adhesion and proliferation of melanoma cells was also studied.

**Results:**

Mice given one single intravenous injection of LPS 6 hour prior to cancer cells significantly increased B16M metastasis in BM compared to untreated mice; however, administration of oral celecoxib reduced BM metastasis incidence and volume in healthy mice, and almost completely abrogated LPS-dependent melanoma metastases. *In vitro*, untreated and LPS-treated murine and human BMSC-conditioned medium (CM) increased VCAM-1-dependent BMSC adherence and proliferation of B16M and A375M cells, respectively, as compared to basal medium-treated melanoma cells. Addition of celecoxib to both B16M and A375M cells abolished adhesion and proliferation increments induced by BMSC-CM. TNFα and VEGF secretion increased in the supernatant of LPS-treated BMSCs; however, anti-VEGF neutralizing antibodies added to B16M and A375M cells prior to LPS-treated BMSC-CM resulted in a complete abrogation of both adhesion- and proliferation-stimulating effect of BMSC on melanoma cells. Conversely, recombinant VEGF increased adherence to BMSC and proliferation of both B16M and A375M cells, compared to basal medium-treated cells, while addition of celecoxib neutralized VEGF effects on melanoma. Recombinant TNFα induced B16M production of VEGF via COX-2-dependent mechanism. Moreover, exogenous PGE2 also increased B16M cell adhesion to immobilized recombinant VCAM-1.

**Conclusions:**

We demonstrate the contribution of VEGF-induced tumor COX-2 to the regulation of adhesion- and proliferation-stimulating effects of TNFα, from endotoxin-activated bone marrow stromal cells, on VLA-4-expressing melanoma cells. These data suggest COX-2 neutralization as a potential anti-metastatic therapy in melanoma patients at high risk of systemic and bone dissemination due to intercurrent infectious and inflammatory diseases.

## Introduction

A significant proportion of cancer patients with no clinical evidence of systemic dissemination will develop recurrent disease after primary tumor therapy because they already had a subclinical systemic spread of the disease [1]. Bone marrow (BM) is a common site of occult trafficking, infiltration and growth of blood-borne cancer cells, and their metastases are a major cause of morbidity [2]. Not surprisingly, circulating cancer cells infiltrate BM tissue and interact with hematopoietic microenvironment at early stages of progression for most of cancer types [3]. Subsequent invasion and growth of metastatic cells at bony sites appear to be facilitated by TGFβ [4] and hematopoietic growth factors [5,6], tumor-associated angiogenesis [7,8] and bone remodeling [9]. Thus, the understanding of complex interactions between cancer and bone cells/bone marrow stromal cells leading to these prometastatic events is critical for the design of an organ-specific therapy of bone metastasis.

The BM colonization of metastatic tumors, both of epithelial and non-epithelial origins, is promoted by inflammation [6,10]. Proinflammatory cytokines released by cancer cells [11] and tumor-activated BM stromal cells [12] increase cancer cell adhesion to bone cells [13] and bone resorption [14,15]. In addition, PGE2 induces VEGF [16] and osteoclast formation [17] in preclinical models of bone-metastasizing carcinomas, suggesting that inflammation can lead to tumor-associated angiogenesis and osteolysis with the involvement of cyclooxygenase-2 (COX-2)-dependent mechanism. Interestingly, COX-2 gene is constitutively overexpressed by most of human epithelium-derived malignant tumors and plays a role in their growth [18-20] and metastases [21]. Human melanoma, a non-epithelial tumor characterized by a marked inflammatory stromal response and osteolytic metastases, also overexpresses COX-2 gene [22], which may be correlated with the development and progression of disease [23]. Moreover, as shown by immunohistochemistry, COX-2 expression in primary melanomas is restricted to melanoma cells and significant correlation between immunohistochemical staining, tumor thickness and disease-specific survival has been reported [24], suggesting that COX-2 is a prognostic marker and a potential therapeutic target, although its role in the complex pathogenic process of bone metastasis is unclear [3].

In the present study, we analyzed the effect of a selective COX-2 inhibitor celecoxib --a 1,5 diarylpyrazole with >300-fold selectivity for COX-2 versus COX-1 [25]-- in a model of generalized BM dissemination of left cardiac ventricle-injected B16 melanoma (B16M) cells [26] into healthy and LPS-pretreated mice, to mimic the prometastatic effects of systemic inflammation [26-29]. Next, we studied the role of COX-2 in the regulation of murine B16 and human A375 melanoma cell adhesion and proliferation in response to primary cultured murine and human BM stromal cell (BMSC)-conditioned media (CM) *in vitro*. Furthermore, the specific effect of exogenous and endogenous BMSC-derived VEGF as mediator of COX-2-dependent melanoma cell adhesion and proliferation was also evaluated *in vitro*.

Our data demonstrate the remarkable contribution of tumor COX-2 to the regulation of melanoma cell adhesion to BMSCs and proliferation in response to BMSC-derived VEGF, and suggest anti-metastatic effects of neutralizing COX-2 in melanoma patients at high risk of bone dissemination.

## Materials and methods

### Drugs

SC-58635 (celecoxib) was provided by Richard A. Marks (Manager, Discovery Res. Adm., GD Searle & Co, Skokie, IL). In addition, Lab Control 1/2 (non-irradiated) Rodent Chao at 1600 PPM and Mod Cert Rodent w/o 16% celecoxib were also provided by GD Searle & Co, Skokie, IL.

### Animals

Syngeneic C57BL/6J mice (male, 6-8 weeks old) were obtained from IFFA Credo (L'Arbreole, France). Animal housing, their care and experimental conditions were conducted in conformity with institutional guidelines that are in compliance with the relevant national and international laws and policies (EEC Council Directive 86/609, OJ L 358. 1, Dec. 12, 1987, and NIH *guide for the care and use of laboratory animals*. NIH publication 85-23, 1985).

### Culture of Cancer Cells

Murine B16 melanoma (B16M) cells from the B16F10 subline, and human A375 melanoma (A375M) cell lines were obtained from ATCC (Manassas, VA) and utilized in the present study. Both cell lines were cultured in endotoxin-free Dulbecco's modified Eagle's medium supplemented with 10% FCS and penicillin-streptomycin, all from Sigma-Aldridch (St Louis, MO). Cultures were maintained and passaged as previously described [29].

### Systemic Dissemination of Cancer Cells via Left-Cardiac Ventricle Injection

Mice (10 per experimental group; experiments performed in triplicate) were anesthetized with Nembutal (50 mg/kg body weight), kept at a warm temperature of 25°C, and the anterior chest wall was shaved and prepared for aseptic surgery by washing with iodine and 70% ethanol. The ribs over the heart were exposed, and a 30-gauge needle attached to a tuberculin syringe was inserted through the second intercostal space to the left of the sternum, into the left ventricle. When blood entered the tip of the needle, 5 × 10^4 ^viable cancer cells in 50 μL HEPES-buffered DMEM were injected. The needle was withdrawn slowly, and the muscle and skin were closed with a single suture. Mice received one single intravenous injection of 0.5 mg/kg bacterial endotoxin lipopolysaccharide (LPS, *E. coli*, serotype O127:B8) or vehicle, 6 h before left cardiac ventricle injection of B16M cells. Then, they were treated with vehicle or celecoxib until being killed on the 15^th ^day postinjection. Celecoxib was supplied daily in the diet at a dose of 500 mg/Kg along all the assays. The following animal groups (120 mice) were used: (a) Vehicle-treated normal mice (10 mice × 3 experiments); (b) Celecoxib-treated normal mice (10 mice × 3 experiments); (c) Vehicle-treated LPS-injected mice (10 mice × 3 experiments); and (d) Celecoxib-treated LPS-injected mice (10 mice × 3 experiments).

### Bone Marrow Metastasis Quantitation

The skeletal system of each mouse was completely dissected. The number of metastatic nodules was recorded under a dissecting microscope (magnification, 10 ×) for each of the following bones: spine (cervical, thoracic, lumbar, and sacral bones), skull (maxilla, mandible, and cranium), thorax (sternum, ribs, and scapula), pelvis (ilium, ischium, and pubis), foreleg (humerus and radius) and hindleg (tibia and femur). On the basis of this inspection, each bone was scored as either containing a metastatic nodule or being free of microscopic tumor. The percentage of bones positive for metastasis was calculated for the total number of mice in each group (metastasis incidence). In addition, metastasis volume was estimated for each bone segment at the time of mouse death. To accomplish this, bones were directly observed under a video-camera zoom (magnification, 10 ×), and the highly contrasted images of bone segments were digitalized. Then, a densitometric program was used to discriminate the black tissue (melanotic metastases) from normal bone tissue and to calculate the percentage of the bone image occupied by metastases. The metastasis volume was then obtained for each bone segment as follows: the number of recorded metastases per bone segment (maximum of 10) was multiplied by the average percentage of surface occupied by metastasis per bone segment (maximum of 100%) and expressed as a relative percentage with respect to a previously defined maximum for each individual bone segment. To avoid subjective influences on the study of metastases, the recordings were made in a blind fashion. Paired and multiple bones were considered as single bone site with the calculated incidence and metastasis development indices including both or all of the bones, respectively, within an animal. Finally, metastasis incidence and volume in LPS-treated mice were expressed as mean increase percentages with respect to control mice and in the case of celecoxib-treated mice, results were expressed as metastasis incidence and volume inhibition percentages with respect to either untreated mice or LPS-treated animals fed with control chow.

### Murine and Human BMSC Isolation, Culture and Characterization

For murine BMSC isolation, femurs and tibias were removed and perfused with 10 ml DMEM. The BMSC-rich effluent was transferred into 25 cm^2 ^culture flasks and maintained for two days at 37°C in a humidified atmosphere with 5% CO2. Once murine BMSCs had spread out on the culture substrate, the culture medium was exchanged and supplemented with 20% horse serum and 200 μg/ml endothelial cell growth factor supplement (ECGS, from Sigma-Aldridch, St Louis, MO), as previously described [30].

For human BMSC isolation, bone marrow aspirates were obtained from patients undergoing bone marrow harvest for autologous bone marrow transplantation, after informed consent. The BM aspirate was immediately diluted in 1:1 in Hanks' balanced salt solution (HBSS) containing 1 Mmol/L EDTA, and passed through a 40-μm stainless steel filter to remove loosely attached hematopoietic cells. The filter was then placed in a 50 ml conical tube and retained stromal elements were resuspended in 5 ml HBSS, followed by the addition of 0.1% collagenase (Worthington Biochem. Co., Lakewood, NC) for 30 min at 37°C. The digested material was filtered through a nylon gauze and centrifuged at 200 *g *for 5 min at room temperature. Then, cells were cultured in 75-cm2 plastic culture flasks in a concentration of 1 × 10^6 ^cells per ml of medium containing alpha-minimum essential medium (GIBCO, Life Technologies, Gaithersburg, MD), 12.5% fetal calf serum (FCS, GIBCO), 12.5% horse serum (GIBCO), 200 μg/ml ECGS, 10-3 M, hydrocortisone sodium succinate (Sigma), 10-2 M beta-mercaptoethanol (Sigma), 10 μg/ml gentamicine and 10 μg/ml penicillin-streptomycin (Sigma). Cultured were maintained in a humid atmosphere at 37°C and 5% CO2.

Murine and human BMSCs were characterized on the 7^th ^or 15^th ^day of primary culture, respectively. To identify reticular and endothelial cell phenotypes, BMSCs were incubated with 10 μg/ml Dil-Ac-LDL (Biomedical Technologies, Inc., Stoughton, MA) for 6 h and with 1 × 10^7 ^FITC-conjugated latex particles/ml (Polysciences, Warrington, PA) for one additional hour. Under fluorescence, light and phase-contrast microscopy, the number of single and double-labeled BMSCs was recorded in randomly chosen microscopic fields (n = 20) at a magnification of × 400. LDL endocytotic BMSCs, which did not take up latex particles (non-phagocytotic), were considered as endothelial cells, while double-labeled cells were considered as phagocytotic reticular cells. Other BMSCs were resuspended, fixed in cold 70% methanol for 30 min, washed and incubated with anti-human von Willebrand factor antibody (Serotec Ltd., Oxford, England) diluted 1:100 in PBS-1% BSA for 30 min at room temperature; BMSCs were then washed and incubated with a FITC-conjugated rabbit anti-mouse IgG antiserum (1:10 diluted in PBS-1% BSA) for 30 min at room temperature. Omission of the primary antibody was used as control of non-specific binding of the secondary antibody.

Once BMSCs had been characterized, they were resuspended and replated at 1 × 10^6 ^cells/well/ml in 24-well plates. Murine and human BMSC conditioned media (BMSC-CM) were prepared as follows: cultured BMSCs were incubated for 30 min with basal medium or 1 ng/ml LPS. Then, cells were washed and incubated with serum-free medium for additional 6 h and supernatants were collected, centrifuged at 1,000 *g *for 10 min, 0.22 μm-filtrated and used undiluted to treat B16M or A375M cells.

### Cancer Cell Adhesion Assay to Primary Cultured BMSCs

Murine and human BMSCs were cultured for 15 days prior to be used in adhesion assays. B16M and A375M cells were labeled with 2',7'-bis-(2-carboxyethyl)-5,6-carboxyfluorescein-acetoxymethylester (BCECF-AM) solution (Molecular Probes, Eugene, OR). Next, 2 × 10^5 ^cancer cells/well were added to 24-well-plate cultured BMSCs and 10 min later, wells were washed three times with fresh medium. The number of adhering cancer cells was determined using a quantitative method based on a previously described fluorescence measurement system [29]. In some experiments, cancer cells were incubated for 4 h with 6 h-untreated or LPS-treated murine or human BMSC-CM before their addition to BM stromal cells. Some murine BMSC-CM were pre-incubated with 10 μg/ml anti-murine VCAM-1 monoclonal antibodies (R&D Systems, Minneapolis, MN) at 37°C for 30 min before their addition to cancer cells. For celecoxib-treated groups, 1 μg/ml celecoxib was added to cancer cells 30 min prior to basal medium (DMEM), BMSC-CMs, 10 ng/ml recombinant murine or human VEGF (R&D Systems, Minneapolis, MN) or 100 ng/ml PGE2 (R&D Systems, Minneapolis, MN).

### Cancer Cell Adhesion Assay to Immobilized Recombinant VCAM-1

Ninety six-well plates were coated with 2 μg/ml recombinant human VCAM-1 (R&D Systems, Minneapolis, MN) at 4°C overnight. Nonspecific binding sites on plastic were blocked by treating the wells with 100 μl of PBS containing 0.5% BSA for 2 h at room temperature. In some experiments, B16M cells were incubated with either basal medium, or two different concentrations of PGE2, 10 and 100 ng/ml (Sigma Chemicals, St. Louis, MO) for 2 h, or with 1 μg/ml celecoxib for 30 min before addition of 100 ng/ml recombinant mouse VEGF (R&D Systems, Minneapolis, MN). In other experiments, A375M cells were preincubated with or without 1 μg/ml celecoxib for 30 min before addition of basal medium, 6 h-untreated or LPS-treated BMSC-CM, and 10 ng/ml recombinant human VEGF (R&D Systems, Minneapolis, MN) for other 4 h. Then, B16M or A375M cells were BCECF-AM-labeled and after washing, they were added (5 × 10^4 ^cells/well) to quadruplicate wells. Then, plates were incubated for 30 min, in the case of B16M cells, or for 60 min in the case of A375M cells, at 37°C before unattached cells were removed by washing three times with fresh medium. The number of adhering cells was determined using a quantitative method based on a previously described fluorescence measurement system [29].

### Cancer Cell Proliferation Assay

Murine and human BMSC-conditioned media (BMSC-CM) were added to 2.5 × 10^3 ^B16M and A375M cells, respectively, seeded into each well of a 96-well microtiter plate, in the presence or not of either 1 μg/ml celecoxib or 1 μg/ml anti-VEGF monoclonal antibody. Control melanoma cells were cultured in the presence of basal medium (DMEM) used in generating BMSC-CM. In some wells, 10 ng/ml recombinant VEGF was added to melanoma cells in the presence or not of 1 μg/ml celecoxib. After 48 h incubation, B16M and A375M cell proliferation was measured using sulforhodamine B protein assay, as previously described [31]. Each proliferation assay was performed in cuadruplicate and repeated three times.

### Measurement of Cytokine Concentration in murine BMSC supernatants

TNFα and VEGF concentration were measured in supernatants from primary cultured BMSC using an ELISA kit based on specific murine TNFα and VEGF monoclonal antibodies as suggested by the manufactures (R&D Systems, Minneapolis, MN).

### Western Immunoblot Analyses

To study COX-2 expression by cultured B16M, basal medium-cultured B16M cells were treated or not for 4 h with 10 and 100 ng/ml recombinant murine VEGF. Then, they were collected in the lysis buffer [300 mM NaCl, 50 mM HEPES, 8 mM EDTA, 1% NP40, 10% glycerol, 1 mM Na3VO4, 0.1 mM DTT, 10 mM NaF and protease inhibitor cocktail tablets, as suggested by the manufacturer (Roche Diagnostics, Mannheim, Germany)]. Same amount of protein from cell lysates were size-separated on 10% SDS-PAGE gel and transferred overnight to a nitrocellulose membrane (BioRad, Laboratories, Hercules, CA). Blots were blocked for 2 h with 5% non-fat milk and then incubated for 1 h with rabbit monoclonal antibody against human COX-2 (Oxford Biomedical Research, Rochester Hills, MI) diluted 1:500 with PBS. Blots were then incubated with peroxidase conjugate anti-rabbit IgG (Santa Cruz Biotechnology, Santa Cruz, CA). Bands were visualized using the Super Signal West Dura Extended Substrate kit (Pierce, Rockford, IL). Equal protein loading in the 10% SDS-PAGE electrophoresis was confirmed by immunoblotting for beta-tubulin expression. Bands were scanned and densitometrically analyzed using the NIH image analysis program for Macintosh to obtain normalized COX-2/β-tubulin values.

To study VCAM-1 expression by BMSCs, basal medium-cultured cells received or not 1 ng/ml LPS for 6 h. Then, they were washed with PBS and disrupted with RIPA buffer (50 mM Tris, 150 mM NaCl, 1% NP-40, 0.5% deoxycholic acid, 0.1% sodium dodecyl sulfate, 2 mM EDTA, 10 mM NaF, 10 μg/ml leupeptin, 20 μg/ml aprotinin, a nd 1 mM phenylmethylsulfonylfluoride). Proteins from cell lysates were immunoprecipitated with 10 μg goat anti-mouse agarose-conjugated VCAM-1 polyclonal antibody (Santa Cruz Biotechnology, Santa Cruz, CA) and blots were blocked and incubated with rat anti-mouse VCAM-1 monoclonal antibody (Serotec Ltd) diluted 1:500 with 5% milk-PBS. Blots were next incubated with peroxidase conjugated goat anti-rat IgG (Santa Cruz Biotechnology, Santa Cruz, CA). Bands were visualized using the Super Signal West Dura Extended Substrate kit (Pierce, Rockford, IL) and were scanned and densitometrically analyzed using the NIH image analysis program for Macintosh to obtain normalized VCAM-1/β-tubulin values.

### Statistical Analyses

Data were expressed as statistical software for MS windows, release 6.0 (Professional Statistic, Chicago, IL). Homogeneity of the variance was tested using the Levene test. If the variances were homogenous, data were analyzed by using one-way ANOVA test with Bonferroni's correction for multiple comparisons when more than two groups were analyzed.

## Results

### Inhibition of Melanoma Bone Marrow Metastasis by Celecoxib

**Figure 1:**
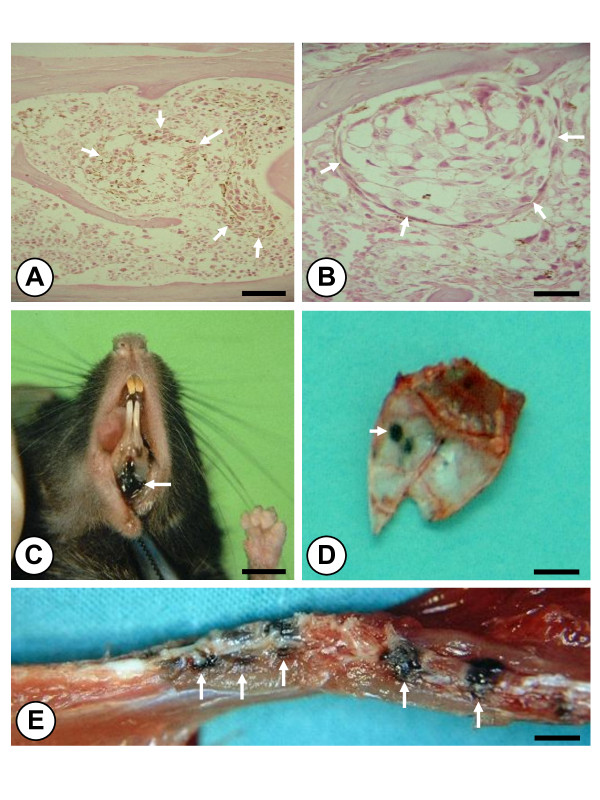
**(*A and B*) Bone marrow micrometastases (arrows) surrounded by red hematopoietic tissue in vertebral bodies on the 10^th ^day after B16 melanoma cell injection (Scale bars: 250 μm in A and 50 μm in B)**. **(*C*) **Gum pigmentation due to mandible metastasis and **(*D*) **skull of a mouse showing a melanotic nodule (arrows) in flat bones on the 15th day following left cardiac ventricle injection of B16M cells (Scale bars: 4 mm); **(*E*) **Compression of the spinal cord due to metastases of B16M cells to lumbar vertebral bodies (arrows) was observed (Scale bar: 2 mm).

Mice developed a mean number of 35 ± 6 macroscopic metastases by day 15 after LCV injection of B16M cells. As previously reported [26], bone was one of the most frequent sites of metastasis in this tumor model. The histological examination of bones by day 10 after cancer cell injection prior to macroscopic development of metastases, revealed subclinical micrometastases limited to the hematopoietic tissue of red BM, which indicates that bone-infiltrating B16M cells specifically colonized extravascular compartments of BM (Figure 1A and 1B). Thereafter, macroscopic metastases occurred in the periphery of flat bones and in the metaphysis of long bones. In addition, metastasis incidence variation among different bone segments (Figure 1C, D and 1E) made it possible to define two bone subgroups: 1) Bones with high metastasis incidence (Table 1), involving the maxilla, mandible, spine, ribs, ilium, humerus, scapula, femur, and tibia; and 2) bones with low metastasis incidence (having 50% fewer metastases), comprising the radius, pubis, ischium, sternum, and cranium.

**Table 1:** Metastasis development in high metastasis incidence bones following Injection of murine B16 melanoma cells into the left cardiac ventricle of mice*

	Metastasis	Average Metastasis
Bones	Incidence (%)†	Development index
Maxilla	76.1	63.2 ± 4.3
Mandible	77.5	63.2 ± 3.9
Tibia	69.6	51.9 ± 3.5
Femur	74.4	40.6 ± 2.7
Spine	68.4	32.5 ± 2.7
Ribs	72.2	26.5 ± 2.9
Scapula	58.3	35.4 ± 2.0
Humerus	73.5	42.6 ± 3.5

*30 mice from 3 independent experiments (10 mice in each experimental group) were cervically dislocated on the 15^th ^day after left cardiac ventricle injection of 5 × 104 melanoma cells in 0.1 ml HEPES-buffered DMEM. *See *"Materials and Methods" section for details.

†Each bone was scored as either containing a metastatic nodule or being free of microscopic tumor, and the percentage of bones positive for metastases was calculated for the total number of bones sites.

The number of recorded metastases per bone segment (maximum of 10) was multiplied by the surface percentage occupied by metastases (maximum of 100) and expressed as a relative percentage with respect to a previously defined maximum for each individual bone segment. Data represent average values ± SD (n = 30).

Paired and multiple bones were considered as single organ sites with the incidence and metastasis development index calculated including both or all the bones within an animal.

**Figure 2:**
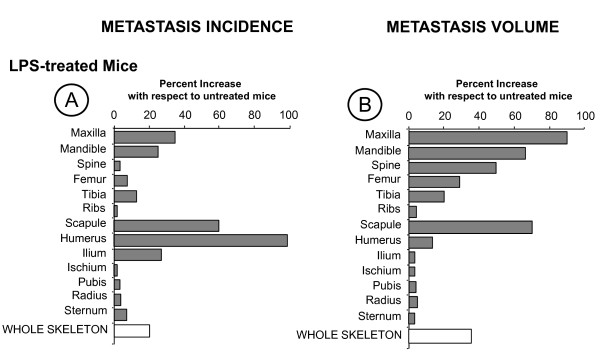
**Effect of LPS on the metastasis incidence (A) and volume (B) of major bone segments of mice injected in the LCV with B16M cells**. Mice (n = 15) were injected intravenously with LPS (0.5 mg/kg body weight). Control mice (n = 15) received the same volume of saline. Six hours later, both mouse groups were LCV-injected with 5 × 10^4 ^B16M cells in 0.1 ml HEPES-buffered DMEM as described in Methods. After 15 days all mice were killed by cervical dislocation and the incidence and volume of metastasis were determined using morphometrical procedures. This experiment was repeated three times. Results are expressed as mean increase percentages with respect to metastasis incidence and volume in control mice.

Mice given 0.5 mg/kg LPS as a single intravenous injection 6 h prior to B16M cell injection exhibited a generalized enhancement of bone metastasis, which significantly (*P *< 0.05) raised the number of bony sites harboring metastases per mouse compared to saline-treated mice (Figure 2A and 2B). However, this prometastatic effect of endogenous inflammation was also bone-specific: 1) LPS significantly (all *P *< 0.05) increased the metastasis incidence and volume in the maxilla, mandible and scapula; 2) metastasis volume, but not incidence, significantly (all *P *< 0.05) increased in the femur, tibia and spine; 3) metastasis incidence, but not its volume, significantly (all *P *< 0.05) increased in the humerus and ilium; and 4) no significant metastasis increase was observed in ribs.

**Figure 3:**
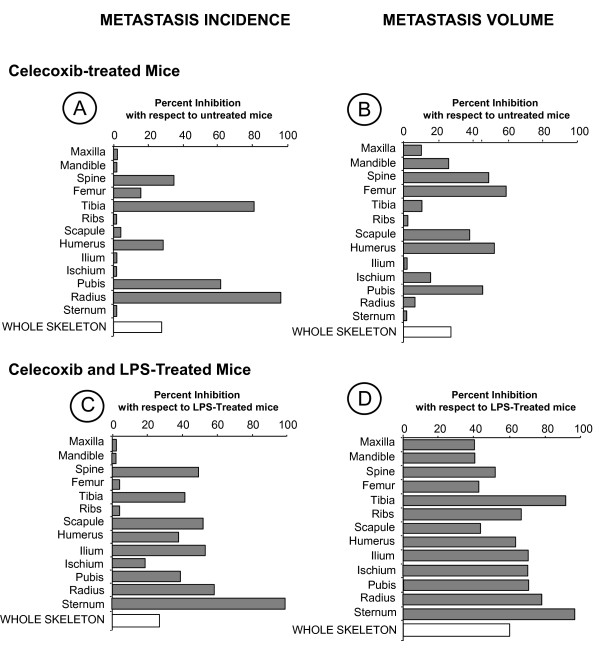
**Inhibitory effect of celecoxib administration on BM metastasis in untreated (A and B) and LPS-treated mice (C and D)**. Mice received either saline or LPS (20 mice per group) 6 h prior to B16M cell injection as above. Ten mice of each group received control chow and the other ten mice received chow containing 16% celecoxib. Treatment was initiated at the time of tumor injection. Mouse killing on day 15 and metastasis assessment was done as above. The experiment was repeated three times. Results are expressed as average metastasis incidence (A and C) and volume (B and D) inhibition percentages determined with respect to animals fed with control chow receiving saline (A and B) or LPS (C and D).

Other mice received either control chow or chow containing 16% celecoxib since the time of tumor injection. Application of this treatment schedule to B16M cell LCV-injected healthy mice significantly (*P *< 0.01) reduced the formation of metastases in several bones. There was a statistically significant (all *P *< 0.05) reduction of metastasis incidence in the spine, pubis, femur, tibia, humerus, and radius, whereas the decrease of incidence in maxilla, mandible, ilium, ischium, ribs, scapula and sternum was not significant in comparison to control mice (Figure 3A). In addition, the metastasis volume dropped significantly (all *P *< 0.05) in most of bones having enhanced incidence of metastases, except for the tibia and radius (Figure 3B). Therefore, an important number of metastases in evaluated bones depended on COX-2-dependent activity under normal physiological conditions. Conversely, celecoxib-unaffected metastases also occurred in several bones, indicating that other COX-2-independent mechanisms also contributed to metastasis.

In mice receiving celecoxib since the time of LPS administration, LPS-mediated enhancement of both metastasis incidence (Figure 3C) and volume (Figure 3D) significantly decreased as compared with LPS-treated mice. This indicates that of the many endogenous factors released in response to LPS, those COX-2-dependent accounted for metastasis-promoting effects of LPS in some bones. However, the fact that LPS-mediated metastasis incidence augmentation did not significantly (*P *< 0.01) decrease in maxilla, mandible, femur and ribs with celecoxib treatment indicates that other COX-2-independent mechanisms were contributing to prometastatic effects of LPS in these bones. Celecoxib also inhibited LPS-induced metastases in other organs, as for example liver, lung, adrenals, and kidney. However, not statistically significant variations of metastasis parameters were observed in heart, testes, brain, skin, and gastrointestinal tract, as compared to untreated controls receiving LPS (data not shown). The vehicle given to mice in the groups used as controls did not significantly alter the incidence or the development index parameters in comparison with the values obtained for normal mice that did not receive any saline injection (data not shown).

### Celecoxib Inhibits Proadhesive Response of Melanoma Cells to LPS-Activated Bone Marrow Stromal Cell-Derived Factors *in vitro*

**Figure 4:**
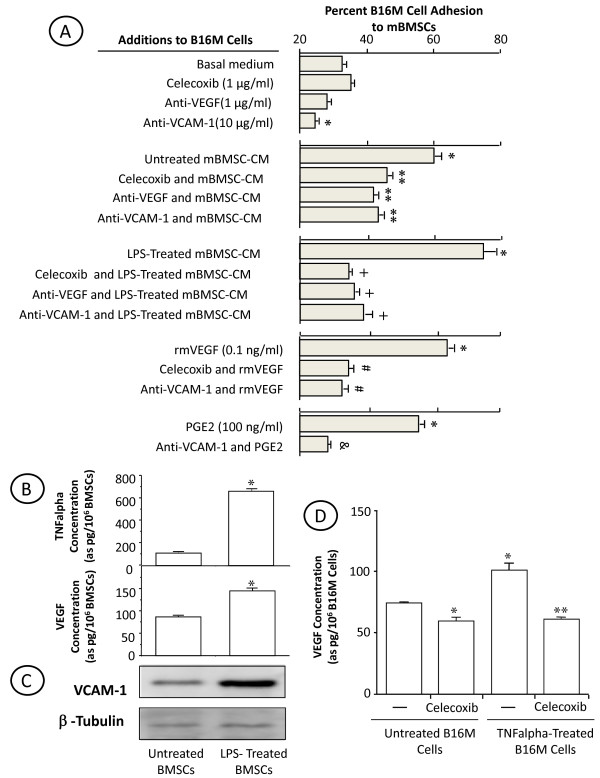
**(A) Effect of celecoxib and anti-VEGF on the proadhesive response of B16M cells to BMSC-CM *in vitro***. Murine B16M cells received 1 μg/ml celecoxib for 30 min and then incubated in the presence of basal medium, BMSC-CM, LPS-treated, BMSC-CM, rmVEGF (10 ng/ml) or PGE2 (100ng/ml) for 4 h. In some experiments, B16M cells received 1 μg/ml murine anti-VEGF monoclonal antibody 30 min prior to BM conditioned media. Once treatments were finished, a B16M adhesion assay to BMSCs was performed. In other experiments, anti-VCAM-1 antibody (10 μg/ml) was added to the cultures of BMSCs 30 min before adhesion assay. Differences were statistically significant cells (*P *< 0.01) with respect to (*) basal medium- or (**) BMSC-CM- or (+) LPS-treated BMSC-CM, (#) rmVEGF-treated melanoma cells or (&) PGE2-treated melanoma cells according by ANOVA and Bonferroni's post-*hoc *test. (B) Effects of LPS on TNFα and VEGF production. Supernatants were obtained from B16M cells incubated 1 ng/ml LPS for 6 h. A competitive enzyme immunoassay was carried out to determine murine TNFα and VEGF concentration. Statistical significance by ANOVA and Bonferroni's posthoc test (*) p < 0.01 vs untreated BMSC. (C) Effect of LPS on VCAM-1 expression by BMSC. BMSC were treated with basal medium and LPS (1 ng/ml) for 6 h. Then, cell lysates were collected and assayed for VCAM-1 and β-tubulin levels by western immunoblot. (D) Effect of celecoxib on TNFα-treated B16M cells. B16M cells received 1 μg/ml celecoxib 30 min prior to TNFα incubation for 4 h (10 ng/ml). Statistically significant by ANOVA and Bonferroni's posthoc test (*) p < 0.01 vs untreated B16M cells, (**) p < 0.01 vs TNFα-treated B16M cells. All data represent media ± SD of 3 separate experiments, each in six replicates (n = 18)

In the next set of experiments, monolayers from short-term primary cultured (two-weeks) murine BMSCs were used to analyze their contribution to the mechanism of B16M cell adhesion under basal and LPS-induced conditions. BMSCs were isolated from two representative bones --femur and tibia--, where LPS-dependent and -independent metastases simultaneously occurred. After two-week culture, majority of BMSCs (97%) showed remarkable DiI-Ac-LDL and OVA-FITC endocytosis, and VCAM-1 expression. Of these, 48% expressed von Willebrand antigen, suggesting their endothelial cell phenotype. The other 52% BMSCs did not express von Willebrand antigen but phagocytosed 1 μm-diameter FITC-latex beads, suggesting their reticular cell phenotype. The 6 h-conditioned medium produced by cultured BMSCs (BMSC-CM) receiving 1 ng/ml LPS significantly (*P < 0.01*) increased B16M cell adhesion to BMSC substrate compared to the adhesion of those receiving untreated BMSC-CM (Figure 4A). In turn, untreated BMSC-CM also significantly (*P *< 0.01) increased adhesion of B16M cells to BMSC substrate as compared to the adhesion of basal medium-treated B16M cells. Therefore, soluble factors from untreated and LPS-treated BMSCs induced the adhesive phenotype in certain B16M cells enlarging the cellular fraction able to interact with BMSCs. More importantly, the pre-incubation of BMSC monolayers with 10 μg/ml anti-mouse VCAM-1 antibody for 30 min prior to adhesion assays abolished adhesion enhancement induced by both untreated and LPS-treated BMSC-CM, indicating that VLA-4/VCAM-1 interaction was mediating the BMSC attachment of B16M cells activated by BMSC-derived factors (Figure 4A).

The role of COX-2 in the upregulation of VLA-4-stimulating activity of BMSC factors on B16M cells was addressed by exposure of B16M cells to celecoxib. Administration of 1 μg/ml celecoxib to B16M 30 min prior to BMSC-CM completely abrogated (*P *< 0.01) adhesion-stimulating activity of both untreated and LPS-treated BMSC-CM (Figure 4A), indicating that BMSC factors upregulated the ability of activated melanoma cells to adhere to BMSCs via COX-2-dependent VLA-4 expression.

Consistent with the strong melanoma cell adhesion-stimulating activity detected in the conditioned media from LPS-treated BMSCs, TNFα and VEGF significantly (*P *< 0.01) increased in the supernatant of LPS-activated BMSCs as compared to untreated BMSCs (Figure 4B). In turn, VCAM-1 expression level also significantly increased in LPS-treated BMSCs, as evaluated by Western blot (Figure 4C).

**Figure 5:**
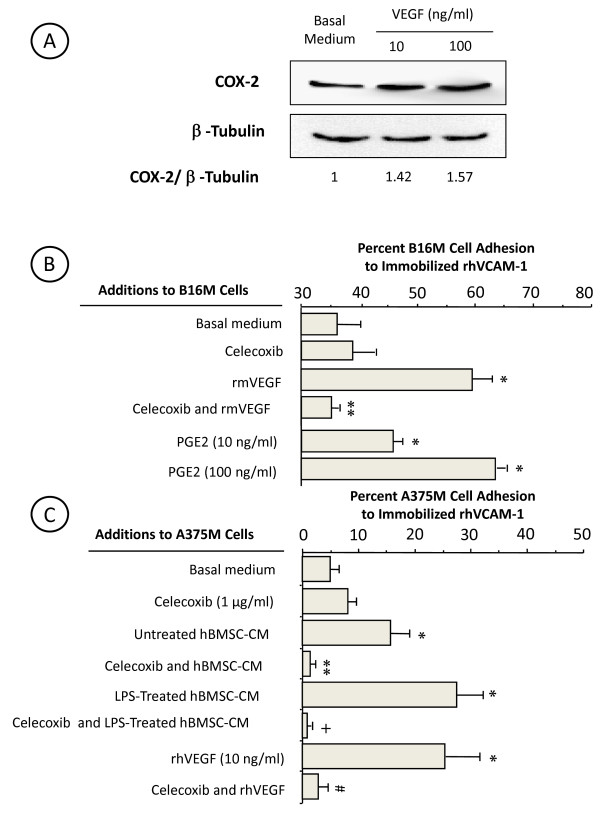
**(A) Representative western blot analysis of COX-2 expression by VEGF-treated B16M cells**. Cultured B16M cells were given 10 or 100 ng/ml murine recombinant VEGF for 4 h. Cell lysates were collected and assayed for COX-2 and β-tubulin levels by western immunoblot. **(B) Effect of celecoxib on the proadhesive response of VEGF-treated B16M cells on immobilized VCAM-1**. B16M cells received 1 μg/ml celecoxib for 30 min and then incubated with 100 ng/ml rmVEGF for 4 h. In other experiments B16M cells were given 10 or 100 ng/ml of PGE2 for 2 h. Then, cell adhesion assay to rhVCAM-1-coated plate was performed. Data are expressed as mean percent of added labeled-cells binding to quadruplicate wells ± SD. Statistical significance by ANOVA and Bonferroni's post-*hoc *test: *P < 0.01 as compared with basal medium-treated B16M cells; **P < .001 as compared with VEGF-treated B16M cells. **C) Effect of celecoxib and anti-VEGF on the proadhesive response of A375M cells to bone marrow-conditioned media on immobilized VCAM-1**. Human A375M cells received 1 μg/ml celecoxib for 30 min and then incubated in the presence of basal medium, hBMSC-CM, LPS-treated hBMSC-CM or rhVEGF (10 ng/ml) for 4 h. Then, cell adhesion assay to a rhVCAM-1-coated plate was performed. Data are expressed as mean percent of added labeled-cells binding to quadruplicate wells ± SD. Statistical significance by ANOVA and Bonferroni's post-*hoc *test: *P < 0.01 as compared with basal medium-treated A375M cells; **P < 0.01 as compared with BMSC-CM-; +*P *< 0.01 as compared with LPS-treated BMSC-CM-treated A375M cells; #*P *< 0.01 as compared with rhVEGF-treated A375M cells.

On the other hand, recombinant murine TNFα (10 ng/ml, 4 h) also significantly (*P *< 0.01) increased by two-fold B16M cell secretion of VEGF, while addition of celecoxib together with TNFα turned down VEGF to basal level (Figure 4D), indicating that TNFα induced VEGF production from B16M cells via COX-2. Interestingly, the addition of 1 μg/ml anti-mouse VEGF antibody to B16M cells together with BMSC-CM (Figure 4A) completely abrogated adhesion-stimulating effect of both untreated and LPS-treated BMSC-CM on B16M cells. Conversely, rmVEGF given to B16M cells at 100 ng/ml for 4 h significantly (*P <*0.01) increased B16M cell adherence to BMSCs, and administration of 1 μg/ml celecoxib to B16M 30 min prior to rmVEGF abolished (*P *< 0.01) proadhesive effects of this cytokine. Neither anti-mouse VEGF antibody nor celecoxib altered basal adhesion rate of B16M cells to BMSC (Figure 4A). Moreover, addition of 100 ng/ml rmVEGF to B16M cells for 4 h significantly (*P *< 0.01) increased their adhesion to immobilized VCAM-1, and 1 μg/ml celecoxib given to B16M cells 30 min prior to rhVEGF abolished their proadhesive effect (Figure 5A). As evaluated by western blot, proadhesive effect of rmVEGF was accompanied by a significant (*P *< 0.05) increase of COX-2 (Figure 5B). Therefore, VEGF from both LPS-activated BMSCs (Figure 4B) and TNFα-induced B16M (Figure 4D) induced B16M cell adhesion to BMSCs via COX-2-dependent VLA-4 expression. Interestingly, addition of exogenous PGE2 (given at 10 and 100 ng/ml) to B16M cells for only 2 h significantly (*P *< 0.01) increased melanoma cell adhesion to an immobilized rhVCAM-1 substrate, which further suggests that VLA-4-dependent adhesion in VEGF-stimulated B16M cells was mediated by COX-2-dependent PGE2 (Figure 5A)

A375 human melanoma (A375M) cells constitutively expressed COX-2 (100% of the cell population) and VLA-4 (50% of the cell population) [32]. Therefore, A375M cells were similarly pre-incubated with untreated and LPS-treated human primary cultured BMSC-CM and their adhesion to an immobilized rhVCAM-1 substrate was also evaluated. Consistent with B16M cell assays, there was a statistically significant (*P <*0.01) increase in A375M cell adhesion to the VCAM-1 substrate (Figure 5C). Celecoxib (1 μg/ml) given 30 min prior to conditioned media of BMSCs completely abrogated (*P *< 0.01) the adhesion-stimulating activity of both untreated and LPS-treated BMSC-CM on A375M cells. Moreover, addition of 10 ng/ml rhVEGF to A375M cells for 4 h significantly (*P *< 0.01) increased their adhesion to immobilized VCAM-1, and 1 μg/ml celecoxib given to A375M cells 30 min prior to rhVEGF once again abolished its proadhesive effect (Figure 5C). Thus, human A375M cells exhibited the same functional response to endogenous VEGF shown in B16M cells, i.e. the COX-2-dependent enlargement of the cellular fraction able to adhere to BMSCs via VCAM-1/VLA-4 interaction.

### Tumor COX-2 Regulates VEGF-Dependent Melanoma Proliferation in Response to BMSC-CM

**Figure 6:**
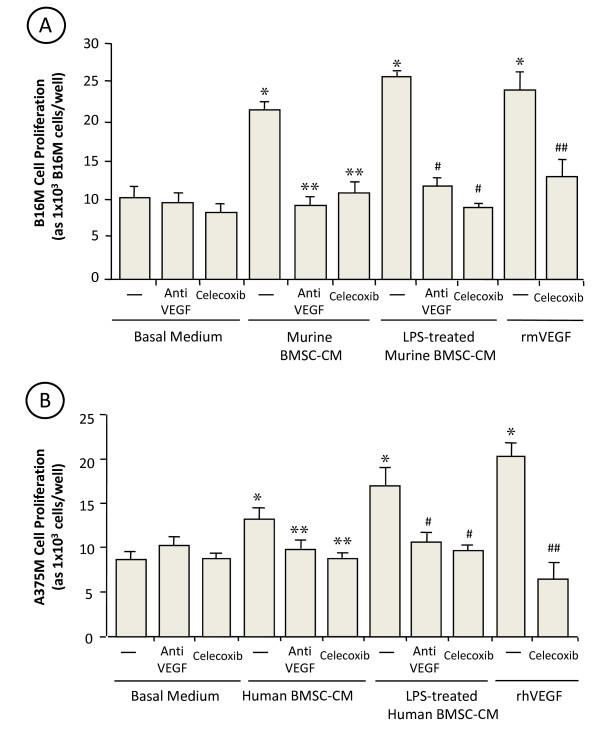
**Effect of celecoxib and anti-VEGF on the proliferation rate of BMSC-CM-treated B16M (A) and A375M (B) cells**. Murine B16M (A) or A375M (B) cells were plated onto 96-well plates at a density of 2,500 cells per well. Some cells received BMSC-CM, LPS-treated BMSC-CM or 10 ng/ml rmVEGF in the presence or absence of 1 μg/ml anti-VEGF monoclonal antibody or 1 μg/ml celecoxib. Control melanoma cells were cultured in the presence of basal medium (DMEM). After 48 h incubation, the number of cells was determined by microscopic counting in 5 different fields per well and by sulforhodamine-101-based fluorimetry as described in Methods. Every assay was done in quadruplicate and repeated three times. Data represent average values ± SD. Differences were statistically significant cells (*P *< 0.01) with respect to (*) basal medium- or (**) BMSC-CM- or (#) LPS-treated BMSC-CM or (##) rmVEGF-treated melanoma cells according by ANOVA and Bonferroni's post-*hoc *test.

Treatment with celecoxib was effective in reducing BM metastasis volume (Figure 3B and 3D), suggesting that COX-2 also contributed to B16M cell growth in the BM microenvironment. As shown in Figure 6A, the conditioned medium from murine untreated and LPS-treated BMSCs significantly (*P <*0.05) stimulated proliferation of B16M cells as compared to basal medium-treated cultures. The ability of untreated and LPS-treated BMSCs to increase B16M proliferation was completely neutralized by celecoxib; however, celecoxib addition directly to B16M cells did not affect their basal proliferation.

As above reported, BMSC-CM contained a basal concentration of VEGF that significantly increased (*P *< 0.01) upon LPS treatment, and B16M cells secreted VEGF in response to BMSC-derived TNFα. Addition of anti-murine VEGF antibody to B16M cells together with BMSC-CM abolished proliferation-stimulating activity of BMSC-CM. The addition of 10 ng/ml rmVEGF also significantly (*P *< 0.01) increased B16M cell proliferation by 2-fold (*P *< 0.01). However, the addition of celecoxib completely abrogated growth-promoting effect of rmVEGF on B16M cells. These findings suggest that VEGF generation in the bone marrow microenviroment from both BMSCs and BMSC-activated B16M contributed to the upregulation of B16M cell growth via COX-2-dependent mechanism.

Finally, A375M cell proliferation also significantly (*P *< 0.01) increased in response to untreated and LPS-treated human BMSCs *in vitro *(Figure 6B). The addition of either celecoxib or anti-human VEGF antibody to A375M cells together with human BMSC-CM also abolished proliferation-stimulating effects. Again, A375M cells given 10 ng/ml rhVEGF also resulted in a statistically significant increase of proliferation that was completely abrogated by celecoxib.

## Discussion

Although over-expression of COX-2 has been associated to the development and progression of numerous human malignancies, melanoma included [18-22], its precise role along the process of cancer cell dissemination and metastasis is still poorly understood. This study provides evidence that B16M cells metastasize in certain bone segments of healthy mice and in the majority of lipopolysaccharide-pretreated mice by COX-2-dependent mechanism.

**Figure 7:**
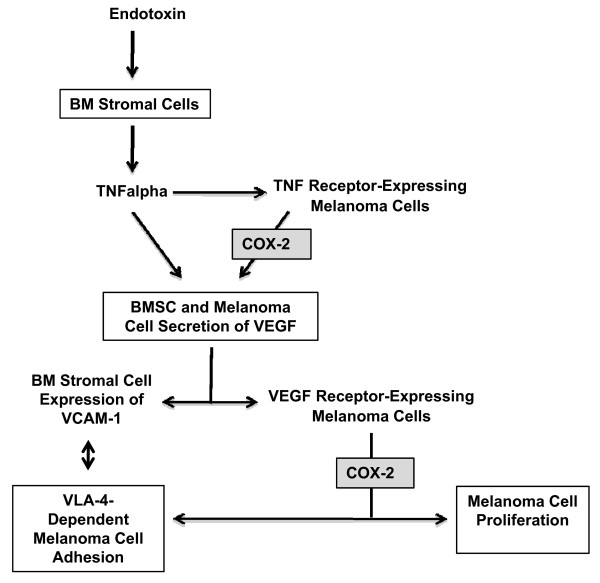
**Model on the contribution of TNFα, VEGF and COX-2 to melanoma metastasis-stimulating effects of bacterial endotoxin-activated bone marrow stromal cells**. TNFα production from LPS-activated BMSCs induces VEGF production and VCAM-1 expression from BMSCs. BMSC-derived TNFα can also stimulate VEGF production from TNF receptor-expressing melanoma cells via COX-2-dependent mechanism. Next, those TNF receptor-expressing and non-TNF receptor-expressing melanoma cells that express VEGF receptors increase proliferation and VLA-4-dependent adhesion to BMSCs via COX-2-dependent mechanism. Therefore, two antimetastatic intervention sites for COX-2 inhibitors may exist in the prometastatic microenvironment generated by endotoxin-activated bone marrow stromal cells.

Because several bidirectional interaction mechanisms occur between BMSCs and cancer cells, which give to the latter a selective advantage for growing in bone [12] and for inducing bone destruction [33], the adhesion and proliferation of melanoma cells in response to soluble factors from primary cultured BMSCs was analyzed. Consistent with *in vivo *data, a COX-2-dependent mechanism was detected, which upregulated both adhesion to BMSCs and proliferation of B16M and A375M cells in response to soluble factors released from both untreated and LPS-treated BMSC *in vitro*. More importantly, VEGF --which is released to the BM microenvironment by both LPS-induced BMSCs [34,35] and TNFα-stimulated melanoma cells [36,37]-- was involved in melanoma-stimulating activities of BMSCs (Figure 7). The mechanism was further supported by experiments revealing COX-2 overexpression in VEGF-treated melanoma cells and enhanced melanoma cell adherence to VCAM-1 induced by exogenous PGE2.

Human malignant melanoma has a propensity to metastasize to bone, where it is exposed to high concentrations of growth-stimulating factors [4-6]. Melanoma cells lodge in the BM since its earliest stage of hematogenous spread [1-3,38] and thereafter can produce osteolytic metastases [39] causing severe clinical complications of the disease. The predilection of melanoma cells for BM was previously demonstrated by using left cardiac ventricle-injected B16M cells [40]. Next, the prometastatic support of BM-derived hematopoietic factors [6] and its stimulation by IL-1 was suggested [41]. Moreover, *in vivo *endogenous IL-1 blockade with IL-1 receptor antagonist revealed that a significant number of BM metastases from B16M are IL-1-dependent, and that all of those developed in LPS-treated mice are IL-1-dependent [26]. Consistent with these data, anti-metastatic effects of celecoxib in the current model suggest that COX-2 --which plays a central role in the mechanisms of inflammation, angiogenesis and bone remodeling [42]--, contributed to the prometastatic activation of melanoma cells in the BM microenvironment of healthy mice and, more remarkably, of mice given endotoxins.

Our data concerning COX-2-dependent metastases are based on single effects of COX-2 inhibitor Celecoxib, and doubts as to a role for off-target actions of Celecoxib may be raised. However, there is ample literature suggesting that COX-2 is indeed involved in the metastatic process [17-25,43,44]. In the present study, both host and tumor COX-2 may be affected by *in vivo *Celecoxib treatment along BM metastasis development. It has been reported that BMSCs adjacent to cancer cells express COX-2 in a murine model of mammary carcinoma [17]. Thus, the possibility that Celecoxib has direct effects on host cell COX-2 should not be discarded. However, in our study such effects might be antimetastatic, especially if we consider that COX-2 inhibition decreases inflammatory and osteoclastic activities that characterize host cell reaction to melanoma cell-derived cytokines [45].

On the other hand, tumor COX-2 also contributes to cancer cell adhesion [46] and proliferation control [47]. In the present work, celecoxib abrogated BMSC-CM-dependent upregulation of B16M cell adhesion to BMSCs and proliferation, suggesting that BM microenvironment activated metastatic behavior of BM-infiltrated B16M cells through tumor COX-2 induction. This was consistent with current data on COX-2 expression by B16M cells and A375M included [22]. However, our study also provides for the first time an indirect evidence that host microenvironment can modulate melanoma COX-2 at specific compartments within a given target organ resulting in a metastatic potential upregulation. This was particularly evident in certain hematopoietic bony sites as for example spine, pubis, femur, and humerus.

An additional finding was that BMSC-derived factors enhanced attachment of B16M cells to BMSCs via VCAM-1/VLA-4 molecular interaction mechanism. Moreover, this proadhesive activation was COX-2-dependent, which suggests for the first time that COX-2 is regulating murine and human melanoma cell adhesion to BMSCs via VLA-4/VCAM-1 mechanism. This was further confirmed by the enhanced adherence to VCAM-1 of PGE2-pretreated melanoma cells. VLA-4 expression confers metastatic properties to human melanoma cells injected into nude mice [32] and has been suggested as marker of poor prognosis in cancer patients, including those affected by melanoma [48]. However, to our knowledge, this is the first evidence on the contribution of COX-2, and more specifically PGE2, to VLA-4-dependent melanoma cell adhesion upregulation.

Our data also demonstrate that VEGF upregulates B16M cell adhesion and proliferation via tumor-COX-2 mediated mechanism, suggesting that VEGF is a microenvironmental factor promoting BM metastasis from VEGF receptor-expressing melanoma cells. VEGF is produced by activated BMSCs and its elevation during BM failure associated to myelofibrosis [49], leukaemia and other neoplastic BM diseases [34] results in BM angiogenesis [35] and mobilization of endothelial and hematopoietic progenitors and stem cells to the peripheral circulation [50]. In the present study, production of VEGF increased in LPS-treated BMSCs and neutralization of VEGF with specific antibodies abolished the effects of BMSC-CM on B16M cells, while B16M and human melanoma cells given recombinant VEGF increased their adhesion and proliferation via a celecoxib-inhibitable mechanism. On the other hand, several human melanoma cell lines [34] and B16M cells [51] also secrete biologically active VEGF. In addition, TNFα --a major inflammatory cytokine released by BMSCs in response to LPS--increased VEGF production from B16M cells via COX-2 (Figure 7). In turn, VEGF appears to induce the adhesive phenotype of B16M cells in a similar way to activated NK cells [52]. This may enlarge the tumor cell fraction able to interact with BMSCs and to growth in the BM microenvironment.

Bacterial endotoxin LPS has been implicated in infectious complications after cancer resection and has been found to enhance metastasis in experimental melanoma [26,31] and other cancer models [53,54]. Both cancer [53] and host cell [54] response to LPS may contribute to LPS-induced metastases. However, in our study, LPS promoted metastasis to certain bony sites, suggesting that prometastatic effects of LPS where mainly due to host cell response to LPS. Not surprisingly, LPS increased VCAM-1 expression and VEGF secretion by BMSCs from hematopoietic bones, which promoted melanoma cell adhesion to BMSCs via tumor COX-2-dependent VLA-4 activation. Therefore, increased circulating endotoxin may be a risk factor for bone metastasis in patients with circulating melanoma cells. However, COX-2 inhibition efficiently blocked LPS-induced BM metastasis and decreased PGE2 production by 4T1 cells *in vitro *[54].

## Conclusions

In the present study we demonstrate that bone marrow stroma cell secretion of VEGF induces melanoma cell adhesion and growth via tumor COX-2-dependent mechanism. This prometastatic mechanism is inducible by bacterial endotoxins, which increase inflammatory cytokine production and VCAM-1 expression from bone marrow stromal cells, and promote bone metastasis, particularly in hematopoietic bony sites. These results suggest that blockade of VEGF effects on metastatic melanoma by COX-2 inhibitors represents a new therapeutic avenue in the prevention and treatment of bone metastases.

## List of abbreviations

The abbreviations used are: COX-2: cyclooxygenase-2; BM: bone marrow; BMSC: bone marrow stromal cell; CM: conditioned medium; B16M: B16 melanoma; A375M: A375 melanoma; LPS: lipopolysaccharide; VEGF; vascular endothelial growth factor; VCAM-1: vascular cell adhesion molecule-1; VLA-4: very late antigen-4; PGE2: prostaglandin E2.

## Competing interests

The authors declare that they have no competing interests.

## Authors' contributions

MV, TC, JJH, OC, CS and LM performed *in vitro *and *in vivo *studies; FVV conceived of the study, participated in its design, coordination, and wrote this manuscript. All authors have read and approved the final manuscript.
